# “It feels like a lightness, and it feels… happy”: Qualitative exploration of transgender and non-binary adults’ understanding of gender euphoria, gender dysphoria, and sexual wellbeing

**DOI:** 10.1080/26895269.2025.2530538

**Published:** 2025-07-12

**Authors:** Daniel A. Griffiths, Fraedan Mastrantonio, Dennis Golm, Heather L. Armstrong

**Affiliations:** School of Psychology, University of Southampton, Southampton, UK

**Keywords:** Transgender, sexual wellbeing, gender euphoria, gender dysphoria, cissexism

## Abstract

**Introduction:**

Considerable research with transgender individuals has centered on gender dysphoria, neglecting the diverse range of transgender experiences and minimizing the importance of opportunities for positive self-identification. However, an emerging body of literature challenges this pathologizing perspective by exploring the concept of gender euphoria, which highlights the affirmative dimensions of transgender experiences and disputes the idea that transgender identities are exclusively defined by hardship and distress. Consequently, this study sought to address the following questions: 1) How do transgender people conceptualize and define gender euphoria as opposed to gender dysphoria?, 2) What are transgender individuals’ experiences with gender euphoria as opposed to gender dysphoria during sexual encounters?, and 3) What additional factors do transgender individuals report as being beneficial or detrimental for their overall sexual wellbeing?

**Methods and Results:**

Drawing from 24 interviews with transgender and non-binary participants and using reflexive thematic analysis, five themes were generated: definitions of gender dysphoria and gender euphoria; cissexist norms and LGBTQ+ community norms; relationship characteristics and sexual wellbeing; embodied gender dysphoria, gender euphoria, and changes through transition; and sexual behaviors.

**Discussion:**

These findings provide insights into gender euphoria among transgender individuals and practical implications for researchers and practitioners. Understanding gender euphoria’s impact on sexual experiences may help enhance the sexual health and overall wellbeing of transgender individuals.

## Introduction

Literature discussing transgender (or trans) individuals’ sexual health and wellbeing has seen increased interest over the past several decades, particularly within medicalized fields (Whitney et al., 2022). Nonetheless, the existing discourse has often been constrained in its discussion of *how* transgender individuals understand their gender identity in relation to their sexual wellbeing, and vice versa. Furthermore, within sexual health literature, transgender individuals’ experiences and behaviors have been largely underrepresented, with the majority of studies focusing solely on cisgender populations (Armstrong, 2020).

Much transgender research has historically focused on individual feelings of gender dysphoria or risk of interpersonal violence (Westbrook, 2021). The term *gender dysphoria*, often defined as “distress that may accompany the incongruence between one’s experienced or expressed gender and one’s assigned gender” (American Psychiatric Association, 2013, p. 451) has become interchangeable with transness. Additionally, gender dysphoria is frequently a diagnostic pre-requisite to access gender-affirming medical care, such as hormone replacement therapy (HRT) or gender-affirming surgeries ([GS]; Dhejne et al., 2016; World Health Organization, 2022). However, many academics and community members alike have pushed back on this overtly negative, deficit-based, and pathologizing narrative of transgender experience in favor of an additional, more positive conceptualization (Benestad, 2010; Bradford et al., 2021; Jacobsen & Devor, 2022). Whilst the term *gender euphoria* can be traced back to 1976 (Jacobsen & Devor, 2022), Beischel et al. (2021) made one of the first research-based attempts to define gender euphoria through the use of an open-ended online qualitative questionnaire, with diverse gender and sexual identity groups, including trans and cis individuals. Through this, gender euphoria was defined as a “joyful feeling of rightness” (p. 281) about gender identity and expression. Despite the minimal academic literature on gender euphoria (Hall et al., 2024), especially regarding sexual experiences, this term has been culturally significant within the transgender community for many years and is often mentioned in nonacademic books, blogs, art, and online discourse (Jacobsen & Devor, 2022).

Similarly, positive sexual health and sexual pleasure have been largely neglected or incorrectly conceptualized within research, with the World Association for Sexual Health creating a Declaration of Pleasure, urging academics and policymakers to prioritize the promotion and understanding of sexual pleasure (Ford et al., 2021). Sexual pleasure and experiences of positive sexuality allow individuals to evaluate the kinds of sexual activities and dynamics most desirable to them (Laan et al., 2021). These experiences of sexual development can often be crucial in supporting an individual’s feelings of sexual wellbeing by expanding the possibilities and boundaries for sexual pleasure, with those who report more pleasurable sexual activities reporting greater levels of desire to engage in such behaviors (Rubin et al., 2019).

Furthermore, *sexual wellbeing* is an often ill-defined term, with little consensus on what factors contribute to an individual’s sexual wellbeing. Lorimer et al. (2019) conducted a rapid systematic review of researchers’ attempts to define sexual wellbeing and indicated that it encompasses individual cognitive-affective factors (e.g., sexual cognitions), interpersonal factors (e.g., relationship satisfaction), socio-cultural factors (e.g., societal stigma), and sexual freedom – and we use this definition for the current research.

Recent evidence suggests that much of what it means to have “sexual wellbeing” or to be considered sexually ‘normal’ has been developed from an overtly cisnormative, heteronormative, and medicalized perspective (Goyal et al., 2022; Özer et al., 2022; Whitney et al., 2022). As such, cisgender and heterosexual experiences have formed the narrative for ‘normative’ sexual functioning. In contrast, Puckett et al. (2023) suggests that transgender individuals’ mere existence challenges pervasive *cissexist* norms—the unconscious and automatic belief that all individuals identify with the gender assigned to them at birth and that this is normal (McGeorge et al., 2021). These norms are based on binary and bio-essentialist views of gender, implying that anyone with a penis and XY chromosomes is automatically considered a man, while anyone with a vulva and XX chromosomes is automatically considered a woman (Barcelos, 2019; Jun, 2018). This leaves little room to acknowledge identities that fall outside of this rigid framework. Finally, a long-standing socio-cultural legacy of pathologizing otherwise normal sexual behaviors, (such as kink, queer sex, masturbation, and sex outside of marriage) has created enduring stigmatizing views of individual private sexual practices (Kılıç Onar et al., 2020; Waldura et al., 2016).

The majority of transgender sexual health literature investigates HIV risk, orgasmic potential following GS, or sexual desire following the initiation of HRT (Bradford & Spencer, 2020). Whilst these are important, pleasure, one of the primary functions and motivations of sex, is often neglected. Additionally, much of the existing literature applies cisgender assumptions of sexual functioning to transgender individuals, despite differences in socio-cultural contexts including stigma and transphobia, and physiological and psychological factors such as HRT and gender dysphoria (Whitney et al., 2022). Making an assumption of cisgender sexual functioning as the norm may bias and limit conclusions made in past research and additionally may reinforce cissexist notions that cisgender sexual functioning is the most appropriate, when these ideals may not be achievable or even desirable for transgender individuals (Whitney et al., 2022).

Sexual experiences may affirm an individual’s gender identity (Stephenson et al., 2017). Partnered sexual experiences and expression of gender identity are both highly social performances – we both *do* sex and *do* gender. Transgender individuals can create a highly affirmative gendered narrative through the language, sex toys/prostheses, and behaviors used during a sexual encounter (Stephenson et al., 2017). For example, a transgender man may choose to refer to their clitoris as their “dick/penis/cock,” or they may choose to refer to their vagina as their “hole,” as an active separation from the stereotypically feminine gendered connotations of a vulva and/or vagina (Jacobsen & Devor, 2022). By engaging in these practices, transgender individuals may not only alleviate feelings of dysphoria but also induce experiences of gender euphoria and joy around and within sexual experiences (Stephenson et al., 2017).

Each stage of medical transition may also provide unique benefits and challenges to an individual’s sexual satisfaction and functioning. In a Dutch study of 221 transgender individuals awaiting care, 39% reported sexual dissatisfaction, despite most reporting that sexuality was important to them (Nikkelen & Kreukels, 2018). Additionally, HRT and GS, or lack of access to these interventions, can also affect sexual function (Holmberg et al., 2019). Androgenizing HRT has been reported to increase sexual desire and satisfaction in transgender men; however, testosterone therapy may cause sexual pain and discomfort due to vaginal atrophy attributed to a suppression of estrogen (Holmberg et al., 2019; Smith et al., 2014). Conversely, estrogen treatment for transgender women has been reported to decrease sexual desire (Holmberg et al., 2019). However, the complex relationships between hormones and sexual desire and pleasure is still contested and ill-understood (Laan et al., 2021).

These challenges may be further compounded as many transgender individuals do not receive adequate sex education in schools due to cissexist, heteronormative, and bio-essentialist curriculums (Gillespie et al., 2022; Nash & Browne, 2019). This may lead to insufficient knowledge about sexual function, health, pleasure, and behavior, and consequently a lack of preparedness for sexual activities (Hobaica et al., 2019). Transgender individuals may also experience inadequate access to sexual health services due to stigma, lack of medical practitioner training on transgender identities, and lack of sexual health literacy (Brookfield et al., 2020). Inadequate access to services may limit an individual’s ability to use preventative strategies such as HIV pre-exposure prophylaxis (PrEP) or post-exposure prophylaxis (PEP), barrier methods such as condoms, contraceptives, or to engage in regular STI testing (Bradford et al., 2019; Gowen & Winges-Yanez, 2014). Socio-cultural norms that restrict transgender individuals’ access to accurate sexual education and sexual health care may lead to negative sexual health outcomes, such as decreased function and satisfaction, higher rates of HIV and other STIs, and shame about their body and sexuality (Haley et al., 2019). Internalizing such cissexist and heteronormative discourses surrounding sexual relationships may further negatively impact transgender and gender-diverse individuals’ self-esteem, sexual wellbeing, and sexual satisfaction, especially if the individual does not align with rigid stereotyped gender roles and sexual scripts (Iantaffi & Bockting, 2011).

Romantic and/or sexual relationships may also interact with transgender individuals’ sexual functioning and satisfaction. External stigma and minority stress against those in cisgender/transgender relationships may lead individuals to conceal their relationships, which has been significantly associated with lower relationship quality (Gamarel et al., 2014; Hendricks & Testa, 2012). A systematic review by Marshall et al. (2020) indicates individual and dyadic variables that may be related to the quality and satisfaction of trans people’s romantic relationships. For example, Platt (2020) and Gamarel et al. (2018) both indicate the importance of relationship commitment on both relationship satisfaction and for protecting against experiences of distress and stigma. Furthermore, Alegría (2010) highlight how communication, strong external social support networks, and self-talk can be protective factors when navigating the challenges of changing sexual identities, uncertainty, and external transphobic stigma within cisgender female and transgender female couples. However, very little research explores how transgender individuals’ experiences of gender dysphoria and gender euphoria within their romantic relationships may impact their sexual functioning and satisfaction. Additionally, much research exploring romantic relationships focuses on the partners of transgender individuals, rather than centering transgender individuals themselves (Pulice-Farrow et al., 2019).

### The current study

Due to the exploratory and descriptive nature of the topic, and the limited existing research, a qualitative interview method was used to explore the following questions:

- How do transgender and non-binary people conceptualize gender euphoria and dysphoria?
- What are transgender and non-binary individuals’ experiences with gender euphoria as opposed to dysphoria during sexual encounters?
- What additional factors do transgender and non-binary individuals report being beneficial or detrimental for their overall sexual wellbeing?

## Methodology

### Participants and recruitment

Recruitment occurred primarily through social media advertising, utilizing platforms such as Twitter/X, Facebook, and Instagram, and LGBTQ+ or transgender-specific support groups. To be included in the sample, participants had to be: (1) 18 years old and over, (2) self-identify as transgender and/or non-binary, and (3) have had previous sexual experiences – including masturbation, intimate touching, oral sex, vaginal intercourse, or anal intercourse. All participants were required to be able to speak English to participate due to language restrictions. Upon completion of the study, participants received a £15 gift card for their time.

### Procedure

Ethical approval was obtained by the University of Southampton Ethics Committee, ERGO-II (ref: 78824). Semi-structured interviews were conducted between February 2023 and April 2023, lasting a mean of 40.32 minutes (SD = 18.97, range: 13:25–81:52). Interviews were conducted through Microsoft Teams or Zoom to increase study reach. All participants provided informed consent before the interview. The first 10 questions were short, closed questions designed to obtain demographic information and build rapport between the interviewer and interviewee. Next, open-ended questions asked participants to describe their definitions of gender euphoria and dysphoria and if/how gender euphoria and dysphoria are present during sexual experiences. Additionally, the researcher asked participants to describe factors, other than gender euphoria or dysphoria, that may impact how satisfying or pleasurable a sexual experience is. Finally, once the researcher reached the end of the topic guide, participants were asked if they had any additional thoughts about gender dysphoria or euphoria during sexual experiences that may not have been covered previously.

Interviews were recorded and subsequently deleted after transcription. Identifying information was omitted from the transcripts.

### Reflexivity

The first author who conducted and analyzed the interviews is a gay, transgender man. These subjectivities and personal experiences were used as a resource in guiding the development and analysis of participants’ data, allowing for a more empathetic and trans-positive perspective. However, as the interviewer and primary analyst, it is vital to recognize the position of researcher when conducting interviews, especially within sexual health research. This positionality may inadvertently create a power dynamic in which participants limit responses due to discomfort and social desirability, especially within sensitive and often stigmatized discussions like transgender sexuality. However, the researcher’s identity as a transgender individual, combined with establishing rapport and using inclusive language, may have aided in mitigating this effect.

To broaden the analytic process and understanding of participants’ data, a second researcher, who is non-binary and well-versed in non-binary sexuality research, helped structure and define themes and subthemes. The meanings of each code and theme were discussed to interrogate differences in interpretations and subjectivities. The inclusion of a non-binary second researcher allowed for a more in-depth and nuanced perspective of different trans identities and experiences throughout the analytic process.

### Data analysis

Reflexive thematic analysis (Braun & Clarke, 2006, 2019, 2023, 2024) was used to analyze participant transcripts in NVivo (QSR International Pty Ltd, 2020). A social constructionist epistemological perspective was adopted when analyzing the data (Byrne, 2022), positioning knowledge as being socially produced, perspectival, and contextual. Further, an inductive (or bottom-up) coding approach was used, meaning analysis was grounded in the data, rather than from the surrounding theory (Braun & Clarke, 2021). Through this reflexive thematic analytical approach, both semantic and latent meanings were highlighted to describe the data and interrogate and capture the contexts and subjectivities of transgender individuals’ sexual experiences, and how they may perceive gender dysphoria and euphoria over different contexts (such as masturbation or partnered/group sex).

The first researcher familiarized themselves with the data by transcribing the interviews from the audio recordings. Transcripts were auto generated using Microsoft Stream and then manually edited by the first researcher to ensure accuracy between the audio recording and transcript. Next, the transcripts were read and re-read with a reflexive journal and notes taken throughout about potential analytic insights. By revisiting this journal throughout the analytic process, this allowed for reflection and questioning of initial assumptions of the data. Codes were then developed to systematically begin to highlight key semantic and latent ideas from the interviews. Candidate themes were generated and reviewed with the second researcher to determine the appropriateness of the codes and themes in line with the data. This process was completed over several sessions to allow for thorough discussion of the data set and generated themes. Additionally, by revisiting themes over different time points, we were able to approach the data with different perspectives, allowing us to refine and finalize the themes for write up.

## Findings

### Participant characteristics

Twenty-four transgender and non-binary individuals participated in this study. The mean age of participants was 25.58 years (SD = 5.7). Whilst the study was open internationally, the majority identified as White British (70.8%). Additionally, participants identified as non-binary (25.0%), trans masc (25.0%), transgender women (16.7%), and transgender men (16.7%), with the remaining four individuals identifying with a specific non-binary label. All identity labels were self-reported by participants and are used as reported with quotes below. Furthermore, most participants identified as bisexual (37.5%) or queer (20.8%) and most were in an unmarried romantic relationship, either monogamous (37.5%) or polyamorous (33.3%); 20.8% were single. Additional demographic information is presented in Table 1.

**Table 1.** Demographic characteristics of sample.

Demographic characteristics	*n* (%)
Gender	
Non-binary	6 (25.00)
Trans masc	6 (25.00)
Transgender woman	4 (16.67)
Transgender man	4 (16.67)
Genderfluid	1 (4.17)
Genderqueer	1 (4.17)
Non-binary woman*	1 (4.17)
Non-binary man*	1 (4.17)
Sexual orientation	
Bisexual	9 (37.50)
Queer	5 (20.83)
Gay	4 (16.67)
Pansexual	4 (16.67)
Lesbian	2 (8.33)
Race/ethnicity	
White/British	17 (70.83)
White/American	4 (16.67)
White/Scottish	1 (4.17)
Asian/Japanese	1 (4.17)
Latinx/Mexican	1 (4.17)
Relationship structure	
Polyamorous	11 (45.83)
Monogamous	10 (41.67)
Unsure	2 (8.33)
Monogamous but flexible	1 (4.17)
Relationship status	
Monogamous relationship	9 (37.50)
Polyamorous relationship	8 (33.33)
Single	5 (20.83)
Engaged	1 (4.17)
Married	1 (4.17)

*Note*

* Participants were asked to self-describe their gender identity.

Most participants had socially transitioned (*n* = 22, 91.7%) – defined here as having disclosed their transgender status to most individuals in their life (including friends, family, work, etc.) and having their changed name and/or pronouns (but not necessarily legally). One participant did not feel the need to change their name and/or pronouns, and the other only disclosed their genderfluidity to others in safe, queer spaces due to fears of interpersonal violence. Two-thirds of participants (66.6%) had started HRT. Furthermore, 29.2% (*n* = 7) reported having top surgery, 4.2% (*n* = 1) bottom surgery, and 12.5% (*n* = 3) other miscellaneous gender affirmative surgeries such as facial feminization surgery or hysterectomy. Full descriptive statistics regarding gender affirmative care are found in Table 2.

**Table 2.** Gender affirmative care steps descriptives.

Gender affirmative care	*n* (%)	
	*Yes*	*No*
Hormone replacement therapy	16 (66.67)	8 (33.33)
Top surgery	7 (29.17)	17 (70.83)
Bottom surgery	1 (4.17)	23 (95.83)
Misc surgery	3 (12.50)	21 (87.50)

### Results of thematic analysis

Five themes were generated to describe transgender and non-binary individuals’ experiences with gender dysphoria and euphoria in sexual experiences. In the first theme, participants defined what gender dysphoria and euphoria meant to them to contextualize their recounts of their sexual encounters. In the second theme, cissexist norms and LGBTQ+ community norms, participants described how wider sociocultural norms of sex and gender impacted their sexual wellbeing and experiences of gender euphoria and dysphoria. Next, in relationship characteristics and sexual wellbeing, participants described how their partners’ attributes and behaviors positively and negatively impacted their sexual experiences and wellbeing. In the fourth theme, participants described embodied experiences of gender euphoria and dysphoria related to their bodily characteristics and others’ perceptions of them. Finally, participants related their feelings of gender euphoria and dysphoria to their experiences of seeking out sexual experiences and/or relationships, and within specific behaviors such as masturbation and kink/fetish. A summary table of themes and subthemes is available in Table 3.

**Table 3.** Summary of themes and subthemes.

Theme	Subtheme
*Definitions of gender dysphoria and gender euphoria*	Gender dysphoria
	Gender euphoria
*Cissexist norms and LGBTQ+ community norms*	Stereotyped cisheteronormative norms
	LGBTQ+ community sexual norms
	Societal [mis]understandings of transgender individuals
	Feeling developmentally behind peers and lack of sex education
Relationship characteristics and sexual wellbeing	
	Trans4trans (T4T) relationships
	Beneficial partner characteristics/behaviors
Embodied gender dysphoria, gender euphoria, and changes through transition	Body dysphoria
	Body euphoria
	Changes through transition
Sexual behaviors	Seeking romantic and/or sexual relationships
	Masturbation
	Kink and Fetish

#### Definitions of gender dysphoria and gender euphoria

##### Definitions of gender dysphoria

Dysphoria was described similarly across participants, who highlighted overtly negative psychological and physiological responses, often related to their own or others’ perception of their gender expression or anatomy. One of the primary ways participants defined gender dysphoria was as a feeling of disconnect between their body and their internal sense of self:

*I’d say like a disconnect between, like, your software and your hardware* (P3, 31 years old, non-binary, American, White)

Additionally, participants reported that gender dysphoria sometimes manifests as physical feelings:

*It’s like a form of like pain or discomfort… I feel like sometimes like there could be like a physical component of it in like…in the same way you like, can feel emotions in your chest* (P7, 20 years old, transgender woman, British, White)

For some participants, gender dysphoria was more social and relative to how others perceive their gender identity and/or presentation:

*For me, gender dysphoria is mostly, like, social dysphoria. So, like I want to be seen, just, generally as a guy, like, even if that’s seen as an inherently trans guy…I don’t care like about passing as cis, just want to pass as like a guy*. (P11, 20 years old, transmasculine, British, White).

Finally, several participants described dysphoria as feeling all-encompassing and underlying most of their day-to-day life and interactions:

*It would be always be there and you can kind of tune it out for a bit by focusing on something or working on something but the moment things were quiet or I was like alone in my head or anything, then, then that the noise kind of became louder and louder, and yeah, just… ever pervasive in the background of my memories and of my life and interactions and stuff before I transitioned.* (P4, 30 years old, transgender woman, British, White)

##### Definitions of gender euphoria

Participants typically described gender euphoria in one of two ways. Some participants described it as an intense feeling of happiness or joy in relation to gender identity and/or expression:

*Gender euphoria feels a lot more, like, solid to me, like, it’s… it’s more tangible and because it, it, it feels like a lightness, and it feels… happy, and it has a, it has a real, like, tangible feeling in my body. Which is why, which is why I realized that it’s a more useful guide than gender dysphoria* (P10, 25 years old, non-binary, British, White)

Other participants described gender euphoria as a feeling of quiet contentment and calmness, rather than a rush of intense positive emotions:

*I don’t experience this sort of explosive, joyful gender euphoria anymore, because I feel that that’s something that’s very common when you’re hitting those big… when you’re early enough transition and everything’s new and exciting…It feels weird to call it euphoria because euphoria has those connotations of really intense joy, but the day-to-day experience of living and being seen and treated as the gender that you are, there’s just like a normality… and I still feel that’s part of gender euphoria. It’s a little feeling that I’m being seen the way that I am by people like me*. (P20, 26 years old, non-binary man, British, White)

Some participants noted that their experiences of gender euphoria could change over time, as it often shifted from a novel, exciting feeling associated with transition milestones to an everyday feeling of quiet contentment. Individuals who had undergone all desired gender transition steps commonly associated gender euphoria with contentment and a decrease in gender dysphoria. Conversely, intense joy was often linked to significant transition milestones like initiating hormone replacement therapy or observing surgical outcomes.

Finally, participants described gender euphoria as a feeling of congruence between gender identity and gender expression and that their transition-related decisions were the right ones.

#### Cissexist norms and LGBTQ+ community norms

##### Stereotyped cisheteronormative norms

Participants were critical of societal gender and sexual norms that dictate that women are expected to be submissive, whereas men are expected to be dominant:

*I don’t really think of, for example, topping or being dominant or being submissive or bottoming as particularly one gender. Like, you know, society would maybe suggest men top, and women are submissive ones, but because I don’t really see that in my head* (P13, 38 years old, non-binary woman, British, White)

The majority of participants highlighted experiences in which they had been expected to exhibit specific sexual behaviors or roles due to their gender assigned at birth and gendered socialization. For example, those who were assigned female at birth and have a vulva may be expected to embody a submissive and receiving role during sex, whereas those assigned male at birth may be expected to behave in a dominant and active role. Such expectations from sexual partners may signal to transgender individuals that they are being viewed as their gender assigned at birth rather than as their gender identity:

*I guess when we’re having sex and they see boobs bouncing and smooth legs, they almost forget, and I have been referred to in ways that, or like people have interacted [with] me in ways that are incongruous with my gender expression, and so then make me feel bad, and that affects my, that affects my kind of emotional wellbeing during sex* (P1, 19 years old, non-binary, British, White)

##### LGBTQ+ community sexual norms

In addition to not feeling as though they fit within cisheteronormative sexual norms, participants highlighted feeling pressure to exhibit specific sexual behaviors to fit in within LGBTQ+ community spaces. This often left participants feeling as though they weren’t queer or gay “enough”:

*I think this is, again, stuff that I didn’t realize until a bit later was like trying to conform within the gay community, like it was very cis gay centric, and it was all about like… it’s just that this whole idea of, like, the stereotype of cis gay men being super sexual and like, just loving dick and like all this stuff, which is fine and great… I think I was so stuck in the whole like, all gay guys care about is dick, and I don’t have one, so I’m useless in this, you know, like community.* (P16, 23 years old, transgender man, American, White)

A common barrier to sexual and romantic relationships for transgender individuals was a lack of education and understanding from cisgender individuals, even within the LGBTQ+ community where individuals may have higher expectations. This often led to individuals being misgendered or having to educate others:

*Everyone’s a product of their environment and their growing up, and a lot of these cis gays who just don’t really know much about trans stuff like that’s not, it’s not their fault, like it’s absolutely fine, but, you know, you can tell sometimes when people are open to things and when people aren’t really and that’s, that, I think, both contributed to my dysphoria, but also just made it quite hard to navigate sex and hook-ups.* (P16, 23 years old, transgender man, American, White)

Some participants stressed the importance of both online and in-person sex-positive queer spaces and venues for them to understand and express their sexuality either by expressing their disappointment about the perceived lack of these spaces or by stating the importance of spaces they had found, especially to destigmatize and understand their own sexuality:

…*a lot of lesbian communities online are quite… not necessarily based around purity culture… I guess like [the] romantic side of things, whereas it’s very difficult to find people talking about, you know the sex that lesbians have… that can definitely affect, kind of, my approaches towards sex and also being able to find other people who also are, kind of, looking to just try things, or maybe try having a one night stand… and, you know, see if having sex with one person, like there might be ways that they have sex that then would make me realise “ohh maybe, like I feel quite euphoric about this” and then being able to implement that*. (P23, 24 years old, non-binary, British, White)

*It was there that I started to go to these queer positive raves and sex parties and when I… like, that was kind of my moment, or my many moments of being like, “wow, all of these boxes I’ve put myself in and these limits I had are totally made-up, trying to, like, grovel for cisgender approval*. (P18, 22 years old, transgender man, American, White)

##### Societal [Mis]understandings of transgender and non-binary individuals

Participants highlighted how societal stigma that portrays trans and queer sex as particularly perverse affects their sexual wellbeing. Such negative perceptions and stereotypes may therefore impact their sexual health, self-esteem, and mental wellbeing:

*It can often be quite hard to talk about sex stuff, because of the shame, because like, as queer people, like, you’re told that your sexuality is like horrible, and disgusting, and hellish* (P11, 20 years old, transmasculine, British, White).

Additionally, several participants highlighted feeling fetishized and objectified by potential sexual partners due to their transgender identity:

*It’s definitely felt like people were, were interested in me for very much like one part of my body, and it was kind of just, yeah, it felt quite objectifying I guess*. (P16, 23 years old, transgender man, American, White)

Finally, the fear of transphobic violence, particularly in intimate or sexual settings, was frequently highlighted by participants. Such concerns about physical safety and potential harm often lead to sexual avoidance or the inability to fully engage in intimate relationships:

*So like there is always that risk, or like cruising in parks and in bathrooms is actually like a critical part of gay history and both as a method of survival and sexual expression in the face of persecution but also just in community and culture building and then just it’s fun, and I will always be too afraid to go do that, because if I am in the middle of, like, a park at night and in a secluded area and someone doesn’t like the fact that I’m trans, there is like nothing I can do about it* (P18, 22 years old, transgender man, American, White)

##### Feeling developmentally behind peers and lack of sex education

Several participants expressed that due to a lack of opportunities for LGBTQ+ romantic/sexual experiences growing up and a lack of appropriate LGBTQ+ focused sex education in school and home-based settings, they felt underprepared and inadequate compared to cisgender heterosexual peers:

*I felt like I was the only queer person in a lot of spaces and they had boyfriends and tons of boyfriends, and I didn’t experience that, and I had not experienced that, so it was like very alienating in a sense because, you know, boys didn’t perceive me like a potential interest, and with girls I didn’t share the same things, so I never like really understood why* (P9, 24 years old, genderqueer, Mexican, Latinx)

*I think it’s partly from whenever we, kind of, did sex education in schools, it was always very heteronormative, didn’t really touch on the existence of trans people whatsoever, and only a little bit kind of talked about why same sex relationships and how they might look like. So, it was never something I necessarily knew how to talk about*. (P23, 24 years old, non-binary, British, White)

Due to this inadequate sex education, participants stated that much of their education about sex came from erotic media such as porn or fanfiction. Through this, participants were able to learn some of their own sexual preferences:

*I engaged a bit in sort of like… a bit in like gay male porn, but it was mostly straight porn that actually made me go “actually, I kind of want to be on the other end of it”, but I feel better, you know, taking on like the masculine role in straight porn, and it was like, “yeah, actually I would prefer that”* (P21, 20 years old, transgender man, British, White)

#### Relationship Characteristics and Sexual Wellbeing

##### Trans4Trans (T4T) relationships

A majority of participants highlighted a preference for intra-community dating and having sexual/romantic relationships with other transgender individuals. A common driver for this was the assumed shared understanding of transness:

*I only ever have sex with people who are also trans, so they have a vague understanding of what that entails, it’s not necessarily people who are trans who have transitions that are similar to mine, but just not really any cis people*. (P23, 24 years old, non-binary, British, White)

Other transgender individuals were often viewed as safer due to risks to both their physical and emotional safety, including interpersonal violence or feelings of gender dysphoria:

*Luckily, as I said, because I only sleep with other trans people, I’ve not really had to worry about being referred to as, like, a girl or a woman during, because I know that would be, I would feel very dysphoric about that, but I specifically avoid being put in that kind of situation*. (P23, 24 years old, non-binary, British, White)

Participants also reported feeling more euphoric about their gender when having sex with other transgender individuals:

*Definitely in the past when I’ve had sex with other trans people. That’s something that really makes me feel a sense of euphoria, maybe not specifically about my gender, but about being trans in general*…*we were also having sex with other non-binary and trans people, and having those interactions makes you feel so much better about your gender and about being trans in general because you… you just sit there and you go “oh, God, thank God we’re all trans”, like “thank God that you made trans people like how beautiful are we*? (P19, 24 years old, non-binary, British, White)

However, several participants highlighted some potential downsides of dating within the transgender community. This included interpersonal conflict in close queer communities or T4T relationships being idolized as the ideal relationship dynamic for transgender individuals:

*I think the, the downsides of kind of seeking out interactions with trans people is that, the thing about it being kind of messy, where it feels like… I like dating T4T, but sometimes it feels a little bit incestuous just with like how close the community is and it feels like you can’t ever really make something sufficiently private I guess*. (P17, 24 years old, transmasculine, British, White)

##### Beneficial partner characteristics/behaviors

Several key partner traits and behaviors were described by participants to be important for their experiences of gender euphoria and positive sexual wellbeing. Participants highlighted how having a sex-positive partner and/or environment aided in them feeling comfortable expressing their sexuality:

*My partner who I’ve been with since then, has always been very open about that kind of stuff [sex], so that has helped me not feel like it’s an uncomfortable topic to talk about*. (P23, 24 years old, non-binary, British, White)

Participants additionally expressed the importance of partners not only understanding transgender identities but also being supportive during changes or fluctuations in gender identity and/or presentation:

*So my partner’s a cisgender guy and him being responsive to my gender expression at the time is something that makes me really feel… it makes me feel comfortable and makes me like really, really happy… He’s very responsive to that and that’s something that makes me feel seen and it makes me feel, yeah, very emotionally and sexually happy at that time because I’m being referred to in the way that I want without having to say anything because we are so in tune in that way.* (P19, 24 years old, non-binary, British, White)

Trust and intimacy were also commonly highlighted as being important aspects of sexual and romantic relationships, especially in the face of dysphoria:

*I think, like, in the context of sex, but not itself, sexual intimacy, is really useful from like a gender perspective, so like, you know, cuddling or like aftercare or before, I find that’s really helpful in terms of like, maintaining my connection with my body, which I quite often try to avoid because I’m not a huge fan, so that that sort of non-sexual physical intimacy around a sexual encounter is very grounding*. (P8, 26 years old, non-binary, British, White)

Another beneficial relationship characteristic discussed by participants was the ability to openly communicate with their partner. This was important for discussing their preferences for the language and touch used for their anatomy to aid in reducing feelings of dysphoria and increase euphoria. Participants reported that general communication regarding sexual wants and sexual health status/testing was an important ability for their partner/s to have:

*My first girlfriend who was trans, talked me through the beginning of our relationship and we actually sat down together and we kind of, we went through our sex toy bags and then we talked about our bodies of kind of what we do like and what we don’t like, but also names for parts of our bodies, and I just thought that was so helpful, not just from a “I don’t want to call their genitals the wrong thing and give them gender dysphoria”, but just also “what kind of sex do you like?” and like, “do you actually like having your breasts played with?”* (P13, 38 years old, non-binary woman, British, White)

#### Embodied gender dysphoria, gender euphoria, and changes through transition

##### Body dysphoria

Participants frequently reported experiences of gender dysphoria during sexual encounters, which made it difficult for some participants to engage in or enjoy sex. This was often related to specific body areas or discomfort due to their physiological response to sex, such as a feeling of wetness:

*It just kind of felt like people were touching a part of my body that didn’t… wasn’t actually a part of my body like, like, it was just this thing that was alien and I didn’t want anything to, kind of, do with it. And… yeah, I would say dysphoria during sex is a lot of like dissociation from my body and feeling like a part of my body isn’t really mine*. (P17, 24 years old, non-binary, British, White)

Such feelings of body dysphoria often led to participants expressing a desire to have different anatomy. Additionally, participants frequently used phrasing such as “I wish” when discussing this topic, highlighting their desire for change, even when physically not possible.

*I used to have so much dysphoria around, like, not having, like, a cis man’s penis essentially…it wasn’t what I had, it was like the absence of that, and it was this whole thing of, like, I wish I could jerk off like this, I wish I could cum like this, I wish, you know, all these things that I wish I had, but I didn’t*. (P16, 23 years old, transgender man, American, White)

Some participants reported feelings of dysphoria while using sex toys, particularly the use of strap-ons as the lack of sensation reminded them of anatomy they do not possess:

*One thing I thought about in regards of I guess euphoria versus dysphoria was the use of like strap ons and stuff during sex, because it was something like, a friend recommended as like a euphoria thing, and I found it to be very dysphoric because it was very much like, “oh, this is a thing you don’t have, great.”* (P22, 26 years old, transmasculine, Scottish, White)

Whilst some participants reported feeling gender dysphoria when using sex toys, others reported the use of sex toys as an extension of self for gender euphoria and sexual pleasure:

*There is like a particular experience that I can think of that I kind of identified as a bit of a gender moment where I was wearing a strap on and was kind of like fairly like passively, um…like someone else was fucking the strap on while I was wearing it, but I wasn’t like…um, I wasn’t fucking them, and then I orgasmed …the, like, sex act that was happening, um, it almost like put me in the position of, like, a different gender in my mind, which I think like felt like a moment of euphoria*. (P5, 25 years old, non-binary, British, White)

Most participants who could become pregnant expressed a fear of pregnancy. To avoid this, many stated that they avoided vaginal sex, used some form of birth control, or were seeking/had a hysterectomy. Another participant reported dysphoria regarding not being able to get someone else pregnant:

*On the flip side of that, the fact that even if I wanted to, I could never get somebody pregnant. That can… not during sex, because like I don’t, I don’t want to have any children in that sense, but it’s more of a mental thing of even if I wanted to, I couldn’t*. (P23, 24 years old, non-binary, British, White)

Participants also reported feeling dysphoric about how their voice sounds during sex due to a higher pitch to their usual speaking voice:

*I sometimes hate how I sound, like the noises that I make, especially if they’re more high pitched. The difference is more obvious to me now I’ve been on testosterone and my voice is lower in general*. (P8, 26 years old, non-binary, British, White)

Several participants reported using clothes such t-shirts or chest compression binders to help cover body parts they felt dysphoric about during sexual encounters:

*I haven’t had top surgery yet, that is a big one because I’m very uncomfortable with that area of my body in general, so that’s not something that I can, kind of, I guess allow to be factored in when I’m having experiences with people because it’s, it’s just a very established thing from the start, like “my top stays on, don’t touch me there*.” (P23, 24 years old, non-binary, British, White)

Participants highlighted how they avoided sex before transition or when their dysphoria was high due to feelings of discomfort:

*When I’m feeling really dysphoric, cause all of my dysphoria is in my bottom half, I won’t really masturbate as much as normal when I’m in a heavy dysphoria period because I don’t even, I don’t even wanna think about having a vulva, I don’t wanna think about having a clitoris, I don’t wanna think about getting wet, I don’t wanna, I don’t wanna think about any of it because it’s all too overwhelming.* (P19, 24 years old, non-binary, British, White)

Conversely, five participants reported not feeling bottom/genital dysphoria and had no problems using their anatomy during sexual experiences:

*Well, I don’t consider myself to experience dysphoria relating to my genitals, I don’t think I have bottom dysphoria … So sex in itself, masturbation, hasn’t really been a problem for me, it’s just doing something that feels good*. (P8, 26 years old, non-binary, British, White)

##### Body euphoria

Alternatively, many participants reported thought processes and behaviors that helped them feel gender euphoria during sexual experiences. First, seeing different genitalia and secondary sexual characteristics as not inherently gendered was a useful tactic highlighted by participants to alleviate dysphoria and help induce euphoria:

*…there are millions of different types of trans bodies… so it, it does really make you think, like, it almost makes you degender your own body because it’s like “this person identifies the exact same way as me and they have got completely different body shape than me. They haven’t got their tits, they’ve got their tits, they’ve got bottom growth, they haven’t got bottom growth. They’ve got a little bit, like you’ve got body hair, you’ve got hair on your butt but you haven’t got it on your chest, you’ve got chest and on your butt” like it’s so… umm… it, because it’s so vastly different, it makes you feel better about your own expression*. (P19, 24 years old, non-binary, British, White)

*I’m a girl with a penis. I mean, masturbating doesn’t have to involve penis, or I do other kind of masturbating too. But, if it does involve penis, that’s a girl with a penis beating her meat, that’s, that’s like, correct gendered in my opinion, yeah*. (P15, 27 years old, transgender woman, Japanese, East Asian)

Participants also described sexual behaviors that help make them feel more affirmed in their gender. However, several participants highlighted conflicting feelings between enjoying stereotypical gender roles for affirmation and being critical of these roles being enforced by societal standards:

*Generally just like… that does also tend sometimes it, like, the giving roles do tend to lean towards like, you know, dominant top sort of roles that are, you know, I find them quite affirming for me, and I know that is a stereotypical masculine thing that maybe doesn’t need to be, you know, stereotyped in that way, but it’s good for me, I lean into it so I don’t know.* (P21, 20 years old, transgender man, British, White)

Several participants highlighted how porn featuring trans people and/or individuals of the same gender as them was affirmative of their gender and attractiveness as a transgender individual:

*Genuinely hand on heart, I watch a lot of trans orgy porn, a lot of trans orgy porn. And that’s something, like that is linked to my masturbation, that makes me feel good, for the exact same reason that having sex with trans people is, because it’s like, look at all these bodies, look at how different they are and look at how attractive I find all of them and that’s, that is, that makes me feel good about myself*. (P19, 24 years old, non-binary, British, White)

Whilst some participants reported using clothes to hide areas of their body that caused discomfort, others reported using clothes to highlight areas they enjoy or that make them feel good in their gender to feel gender euphoric:

…*my partner got me some nice underwear I wanted, and I was… we were on holiday and I brought those along and I wore those and, and before sex, and you know, they came in and I was there with nice underwear on… and I dunno, yeah, that was a nice kind of moment where I just felt very myself and very feminine*. (P4, 30 years old, transgender woman, British, White)

Finally, using the correct gendered language themselves or by a partner was often an important element for participants to feel gender euphoria during sexual experiences. Many participants wanted explicit gendering:

*I hooked up with this girl once. I think it was about a year ago now. She was a cis girl, but she was queer, and like she, she was like doing, she was like calling me like ‘king’ and stuff and all the good pet names and I was like, “this is really nice to, like, hear, especially from a cis person.*” (P11, 20 years old, transmasculine, British, White)

However, one highlighted how excessive gendering was often an uncomfortable experience as he was used to being correctly gendered in his day-to-day life:

*So because like… it’s not new or exciting for me to be called what I am. I know who I am. It would be just weird if somebody tried to affirm me by like, I don’t know, repeatedly using my name, or pronouns, or gendered language in that kind of way.* (P20, 26 years old, non-binary man, British, White)

##### Changes through transition

Those who had begun HRT or had undergone GS commonly reported noticeable changes to their sexual wellbeing and function. Many participants highlighted how physiological changes from HRT (e.g., bottom growth (testosterone) and breast growth (estrogen)) aided in their sexual wellbeing:

*With one partner, they pointed out, like, boob growth, it’s like, look, you’ve actually got some, which is after eight months on hormones is like “oh that’s amazing” or like pointing out the shape of my hips, like, that I’ve actually got some developing and things like that. So, if other people are noticing the changes that I’ve kind of seen very subtly over like the eight months, it’s really nice that someone else can see those as well*. (P2, 19 years old, transgender woman, British, White)

Some participants highlighted how HRT has changed sexual function, for example causing difficulty maintaining an erection on estrogen, or increased sexual desire on testosterone:

*Erectile function so like… staying erect for a long period of time, it’s just something like… So they’re trying to find protection in a drawer or something… and if that takes time, that can be like, hang on, let’s go back a few steps before we use that*. (P2, 19 years old, transgender woman, British, White)

Those who had top surgery or a hysterectomy highlighted feeling able to engage in more varied types of sex that they were not comfortable doing before. Further, one participant recalled her experiences getting a vaginoplasty and the time it took to become used to her new genitalia:

*So after GRS [gender-reassignment surgery], penetration felt very clinical and, you have to… you have these dilators that you have to use, and, they’re very like, they’re just like straight glass rods that you use in a very clinical way and very systematic way for the first like, especially the first six months or really the first year after GRS and so yeah, I just… It took me a while to kind of feel, yeah, enjoy penetration in that way*. (P4, 30 years old, transgender woman, British, White)

Finally, participants who had undergone all desired gender-affirming treatment plans experienced a reduction in gender dysphoria and felt more able to enjoy their sexual experiences:

*I’m not really thinking about like, how do I feel in my gender in this moment? It’s… but like, to be fair, I think that is a marker of like I am better in my body in general, like I don’t feel the need to think about it anymore*. (P22, 26 years old, transmasculine, Scottish, White)

#### Sexual behaviors

##### Seeking romantic and/or sexual relationships

Several participants reported using dating apps or websites to find potential romantic and/or sexual partners. This was often a preference as apps were seen as easier and safer due to the increased physical distance between individuals:

*I’ve felt really comfortable with this, like cyber texting and stuff like, you know, sending nudes and stuff like that, that allows me to not necessarily put myself in maybe like a place of danger of sorts*. (P9, 24 years old, genderqueer, Mexican, Latinx)

Additionally, some participants highlighted a preference for casual sex to fulfill sexual needs:

*I would say a little compulsive where I would be like, “well, it’s time to go to the club again because I haven’t hooked up with anyone in a week and I want that” or “it’s time to get back on Grindr because maybe someone will message me tonight that I want to hook up with*.” (P18, 22 years old, transgender man, American, White)

##### Masturbation

Some participants viewed masturbation as an important and enjoyable aspect of their sexuality, often being described as a key way to connect with themselves and their bodies:

*I’ve been trying to date myself. I’ve been trying to like take myself out for a date, treat myself to really nice food, like cook myself a really nice meal, and like, even as I’m chopping the vegetables, I’m kind of like “this is for you” and just really bring that in and, and sort of like take that into my masturbation practises as well.* (P10, 25 years old, non-binary, British, White)

Some participants enjoyed masturbation to feel sexual pleasure without the social dysphoria they often felt during partnered sexual experiences:

*Partly because like, masturbating’s, like, a very safe place to like, not really focus… like gender, and gender quite often is, like, how are other people perceiving me?… it feels like you can almost… switch off that part, like, what do the acts that I’m doing say about my body or myself?* (P5, 25 years old, non-binary, British, White)

Masturbation was often seen as fulfilling bodily needs and was repeatedly compared to eating when hungry or sleeping when tired. Some participants seemed to view this as a negative reason to masturbate, or viewed masturbation as a lesser act than partnered/group sex:

*If I am masturbating I am usually much more like… I guess, like, fulfilling a physical need more than anything else. I am not someone who like has a bath and lights candles, and like spends 2 hours, like, just like really making love to myself. I’m much more like OK like, like, usually it’s like my partner is away for a week or something and I would like to get off, so I will just like hang out here for 20 minutes. (P22, 26 years old, transmasculine, Scottish*, White)

##### Kink and fetish

Several participants expressed the importance of kink for their sexuality and/or gender expression. Kink was explicitly linked to feelings of gender euphoria as participants reported that kinks such as impact play/spanking allowed them to feel more connected to their body:

*I think I also attribute kink in a way to gender in that I’ve always, always preferred rough sex, and that for me is something that helped me achieve gender euphoria because I’m not being treated like this dainty, tiny, cutesy little thing, I am… It’s like more kind of raw and almost animalistic, over being put on this almost like princess pedestal, like, I really I don’t, that’s something that I don’t appreciate anymore… being treated roughly is something that does bring me gender euphoria, because I’m, I’m, in the nice way of being treated like a big boy that can take it.* (P19, 24 years old, non-binary, British, White)

Participants additionally reported that kink can allow for non-genital related sexual play and fulfillment. This allowed participants to enjoy sexual contact and intimacy with their partner whilst avoiding areas, like genitals, that cause gender dysphoria:

*I enjoy like sensual stuff and, like, impact stuff and rope where there’s kind of like a thing that you’re both doing together that is erotic and intimate whilst not being, like, genital sex, and, like one, it’s just kind of fun to do and two, it gives that like non-genital kind of intimacy.* (P17, 24 years old, transmasculine, British, White)

## Discussion

Through interviews with 24 transgender and non-binary adults, the following questions were explored: (1) How do transgender and non-binary people conceptualize gender euphoria as opposed to gender dysphoria?, (2) What are transgender and non-binary individuals’ experiences with gender euphoria as opposed to dysphoria during sexual encounters?, and (3) What additional factors do transgender and non-binary individuals report being beneficial or detrimental for their overall sexual wellbeing? From these interviews, five themes were generated: (1) definitions of gender dysphoria and gender euphoria; (2) cissexist norms and LGBTQ+ community norms; (3) relationship characteristics and sexual wellbeing; (4) embodied gender dysphoria, gender euphoria, and changes through transition; and (5) sexual behaviors.

### Conceptualizations and experiences of gender euphoria

Gender euphoria, in addition to dysphoria, was generally considered by participants as an important part of understanding trans experiences, highlighting a distinct gap in our current understanding of transness, which is often dominated by experiences of gender dysphoria and distress. Participants typically described gender euphoria in one of two general ways: feelings of intense joy or contentment and calmness. These differences in personal understanding of transgender experiences and gender euphoria may reflect some of the final stages proposed by Cass (1979) and Lev (2004) in their stage models of identity formation and transgender emergence. Whilst Cass’s identity formation model (1979) focuses upon gay/lesbian identity formation, transgender individuals may face some of the same external cisheterosexist pressures whilst moving through a similar process of understanding their gender identity (Lev, 2004). For example, perhaps those who report primarily experiencing gender euphoria as being explosive and joyous are within the fifth stage of this model: *identity pride*. In this stage, Cass suggests that individuals perceive their differences from cisheteronormative society as being positive and may reject the cisheteronormative status quo, leading to feelings of pride and joy in their gay (or trans) identity. Similarly, participants may be reflecting the fifth stage of Lev (2004)’s transgender emergence model: *exploring transition and possible body modification*. Participants who reported these explosive feelings after beginning transition steps or after being correctly gendered may be rejoicing in their ability to break free from these cisheteronormative scrips and may feel the positive social and psychological benefits of being able to express themselves more freely within their bodies and presentations. Alternatively, those who reported feeling gender euphoria as a sense of quiet contentment may be reflecting Cass’s sixth and final stage, *identity synthesis*, or Lev’s sixth stage, *integration: acceptance and post-transition issues*. After gaining social support from both gay and straight (or both transgender and cisgender) individuals, Cass suggests that individuals may begin to integrate their gay identity into the rest of their personhood, such that it is simply one aspect of their individuality, rather than being their defining characteristic. These individuals were often later in their transition and had been living full time as their gender for multiple years, suggesting that the novelty and excitement of first starting their transition goals has now faded and subsided, similar to the reports of several of our participants.

Our findings also strongly align with Wurm et al.’s (2024) qualitative work on minority joy. Notably, our description of differing experiences of gender euphoria being either explosive and joyous or calm and contented relate to Wurm et al.’s (2024) concepts of minority joy and minority peace. Within their study, *minority joy* was described as pleasurable or happiness inducing experiences related to one’s transgender or gender diverse identity, whereas *minority peace* relates to feelings of tranquility or being at rest within one’s transgender and gender diverse identity. These results lend credence to the notion that gender euphoria may be experienced in diverse and multifaceted ways, relating to individuals’ unique psychological, relational, and sociopolitical contexts. Further, Wurm et al. (2024) highlighted the concept of *minority growth*, the experience of personal development, self-awareness, and authenticity throughout one’s transition journey. Whilst we did not highlight this as a specific subtype of gender euphoria, many of our participants described how their gender euphoric experiences were often vital in their understanding of what aspects of their gender identity, expression, and development felt the most authentic and comfortable throughout their transition journeys.

#### Gender Euphoria in Sexual Experiences

Participants reported several factors that impacted their experiences of gender euphoria during sexual encounters. First, degendering or recontextualizing the language used to describe their anatomy may allow individuals to challenge bio-essentialist and cissexist norms that dictate that all individuals with a penis are men and those with a vulva are women (Martin & Coolhart, 2022). Additionally, participants reported the importance of sex-positive queer spaces and seeing similar others as attractive to aid in destigmatizing their sexuality as a transgender individual. Lev (2004) highlighted the importance of transgender individuals seeking information from others with similar narratives and experiences, especially with the emergence of the internet (Cipolletta et al., 2017). Access to supportive online spaces may allow individuals to anonymously explore their gender identity and expression, especially when not feasible in person, allowing them to create a ‘virtual gender’ and be both ‘in’ and ‘out’ of the ‘closet’ at the same time (Cavalcante, 2016; Lev, 2004). This need for anonymous online educational spaces for transgender individuals may reflect the lack of diversity and inclusion within current sex education programs (Bradford et al., 2019). Furthermore, social support within queer spaces can be invaluable for transgender and non-binary individuals, especially in a hostile and anti-transgender social and political context (Ross & Scholl, 2016). Positive social support from others within the same community may aid in allowing the individual to feel less alienated and more positive about their own gender minority identity (Cass, 1979), which may induce feelings of gender euphoria. However, more research is needed to explore if there is a relationship between social support and gender euphoria.

### Relational factors and sexual wellbeing

Participants identified several relationship and partner characteristics that were beneficial and challenging for supporting their sexual wellbeing. First, participants highlighted the need for their partner to be supportive and accepting of their transgender identity. Acceptance and validation of an individual’s transgender identity may help reduce gender dysphoria and increase gender euphoria, both in and outside of sexual experiences with supportive partners (Galupo et al., 2020; Martin & Coolhart, 2022). Furthermore, the process of transition may introduce additional stressors such as minority stress experiences, which may impact relationship quality for both the transgender individual and their partner (Gamarel et al., 2014). Existing within a heteronormative and cissexist societal context was also reported by our participants to negatively impact their self-esteem and sexual wellbeing, often causing them to doubt their legitimacy or attractiveness in their gender identity or gendered body (Iantaffi & Bockting, 2011).

The ability to openly communicate was an important skill participants desired from their romantic and sexual partners. This was especially important for discussing sexual preferences with potential partners, such as language and touch of certain body parts, or activities like kink or fetish (Griffiths & Armstrong, 2024; Martin & Coolhart, 2022). Finally, several participants highlighted their preference for romantic and sexual contact with other transgender and non-binary individuals, often referred to as trans4trans or T4T (Griffiths & Armstrong, 2024). T4T relationships were primarily described as preferred due to a perceived shared understanding of transness and emotional and physical safety, which may also induce feelings of gender euphoria within the relationship.

### Implications

Our findings indicate that trans sexuality is complex and influenced by unique psychological, social, and biological factors. For example, participants highlighted a complicated relationship with stereotyped gendered and sexual norms expected of them due to both their sex assigned at birth and their current gender identity and presentation. Several of these individuals also expressed a level of discomfort around finding stereotyped gendered behaviors or presentations affirming due to their critiques of such norms. However, it is important to recognize the psychological benefits, such as gender euphoria, of affirmative gendered behaviors and presentations that align with an individual’s internal sense of self, whether they align with external societal norms of gender or not. Alternatively, issues may arise when such norms are unwanted and expected of others when they do not align with their gender identity and expression, and thus, may induce feelings of gender dysphoria. Furthermore, it is crucial to acknowledge how transgender individuals have historically faced pressure by medical professionals to conform to stereotyped and binary gendered presentations (Cannoot, 2019; Johnson, 2019). This medicalization of gendered expression, both in order to receive gender affirmative care and to integrate into their gender identity group, may force individuals to present in a way that reflects cultural ideals of binary gender, rather than their own individualized expression of their gender identity (Lev, 2004). As a result, Lev (2004) highlights how both feminist and medical literature has critiqued transgender individuals for their apparent mimicry of sexist gendered norms and for “producing caricatures of masculinity and femininity” (p. 264). However, for many individuals, conformative gender expression is a matter of safety, as being ‘visibly’ transgender may place individuals at substantially higher risk of discrimination, harassment, and abuse (Miller & Grollman, 2015; White & Jenkins, 2017). From this, it is important for clinicians and researchers to ensure they do not impose their own understandings of gender onto those they work with due to the very unique and individual experiences each person, transgender or not, has with their gender identity.

Additionally, our findings indicate the need for inclusive and diverse sex education and sexual health programs that include and promote the sexual wellbeing of transgender and gender diverse individuals. Many of our participants highlighted the lack of appropriate sex education throughout their adolescence, leading them to feel underprepared for their sexual relationships, especially in the context of gender dysphoria and a developing transition journey. However, it is important that clinicians and educators recognize the diversity of gender euphoric and dysphoric experiences within the transgender and gender diverse population. Similar to cisgender individuals, sexual preferences and behaviors are highly individualized within the transgender and gender diverse community. What may be euphoric for some may be dysphoric for others, such as the use of sex toys within partnered or solo sexual encounters. Effective sexual health care and education for transgender and gender diverse individuals requires a holistic, individualized approach that identifies gender-affirming and gender euphoric aspects of their sexual identity to enhance overall wellbeing.

#### Theoretical implications

Our findings contribute to important theoretical implications for our understanding and conceptualization of gender euphoria and gender dysphoria. Instead of focusing solely on gender dysphoria as the primary way to conceptualize the experiences of transgender individuals, we suggest that researchers and clinicians embrace a more comprehensive perspective that acknowledges both gender euphoria and gender dysphoria. Many of our participants highlighted the personal importance of their gender euphoric feelings in navigating their gender transition and their romantic/sexual relationships. However, research has only just begun to acknowledge gender euphoria and its potential psychological and social benefits for transgender and non-binary individuals. Such distinct differentiations may highlight additional complexity in gender euphoria and indicate a need for further research to better understand the nature of gender euphoric experiences. Additionally, our findings indicate the dynamic nature of gender euphoria as it was reported to change the longer individuals lived as their gender. Our findings also indicate that factors inducing euphoria or dysphoria are individualized and contingent on the person, such as the incorporation of sex toys during sexual activities. To go further, researchers should consider whether there are distinct types of gender euphoria, and/or if gender euphoria is experienced differently over the lifespan or depending on personal transition milestones. Finally, researchers and clinicians should work not only to reduce dysphoria, but also to increase feelings of gender euphoria—such that therapeutic outcomes seek to improve overall wellbeing in relation to one’s gender identity, rather than simply reduce gender dysphoria and distress. Doing so may aid in moving away from a deficit-orientated to a strengths-based perspective of transgender wellbeing.

### Strengths and limitations

First, whilst we were able to recruit a handful of international participants, most of our sample identified as White British. This is likely a reflection of our recruitment practices which primarily involved advertising on social media, which may have been unintentionally shared around groups of similar UK-based individuals. Additionally, given the UK’s majority white British population (81.7%; Office for National Statistics, 2022), and the UK’s even smaller population of transgender individuals of 0.5% (Office for National Statistics, 2023), recruiting a diverse sample may prove challenging in the UK. We were, however, able to capture a diverse sample of transgender and nonbinary identities within Britain (Office for National Statistics, 2023). Consequently, we were unable to explore the intersectional complexities of racial/cultural identities in the context of transness and individuals’ experiences of sex. For example, Bowleg (2008, 2013) explored the intersection of black and gay/lesbian identities, and highlighted individuals’ struggles with juggling their different identities, pressures to behave in certain gendered/sexual ways in different community spaces, and racial microaggressions and discrimination within white LGBTQ+ community spaces. Furthermore, our interviews were restricted to being conducted in English only. This greatly restricts the diversity of experiences and identities discussed within our results as our sample is primarily White and Euro-American centric. Findings may differ within other cultural or racial contexts due to accessibility of social/medical transition, sociopolitical factors, or community connectedness (Jessani et al., 2024; Pandya & Redcay, 2021; Tan et al., 2021). Further work exploring cultural factors of gender euphoria and sexual wellbeing within transgender and gender diverse individuals is necessary.

Additionally, the mean age of our participants was 25.6 years old (SD = 5.7), with very few participants over the age of 30, likely due to our recruitment methods being primarily online. Thus, our findings may lack the additional insight and lived experiences of older individuals who may have different perspectives of their gender identity and sexualities compared to our younger cohort, especially regarding experiences of transition later in life. Furthermore, whilst identity integration theories do not posit specific age frames for when an individual will go through each stage, as it is an individualized experience (Cass, 1979; Lev, 2004), young adults may be less stable within their identity compared to those who have been living within their gender identity for many years (Lev, 2004). However, further research regarding identity stability and gender euphoria in those who transition later in life is needed to fully explore this relationship.

## Conclusions

Gender euphoria is an important, but under-researched, area of transgender and non-binary individuals’ experiences. Both gender euphoria and gender dysphoria were cited by participants as important constructs for navigating and contextualizing their sexual and non-sexual experiences. Researchers and clinicians should embrace a more comprehensive and holistic perspective of transgender individuals’ experiences that embraces both gender euphoria and gender dysphoria. Furthermore, new research should continue to explore gender euphoria to unravel its complexities and implications for wellbeing.

## References

[CIT0001] Alegría, C. A. (2010). Relationship challenges and relationship maintenance activities following disclosure of transsexualism. *Journal of Psychiatric and Mental Health Nursing*, *17*(10), 909–916. 10.1111/j.1365-2850.2010.01624.x 21078006

[CIT0002] American Psychiatric Association. (2013). *Gender dysphoria*. In Diagnostic and statistical manual of mental disorders (5th ed.). Washington, DC: American Psychiatric Publishing Inc.

[CIT0003] American Psychiatric Association. (2013). Gender dysphoria. In *Diagnostic and statistical manual of mental disorders* (5th ed.). American Psychiatric Publishing Inc.

[CIT0004] Armstrong, H. L. (2020). Sexual disorders among sexual and gender minority populations. In *The Oxford handbook of sexual and gender minority mental health*. Oxford University Press. 10.1093/oxfordhb/9780190067991.013.13

[CIT0005] Barcelos, C. A. (2019). ‘Bye-bye boobies’: Normativity, deservingness and medicalisation in transgender medical crowdfunding. *Culture, Health & Sexuality*, *21*(12), 1394–1408. 10.1080/13691058.2019.1566971 30762488

[CIT0006] Beischel, W. J., Gauvin, S. E. M., & Van Anders, S. M. (2021). “A little shiny gender breakthrough”: Community understandings of gender euphoria. *International Journal of Transgender Health*, 23(3), 274-294. . 10.1080/26895269.2021.1915223 PMC9255216 35799953

[CIT0007] Benestad, E. E. P. (2010). From gender dysphoria to gender euphoria: An assisted journey. *Sexologies*, *19*(4), 225–231. 10.1016/j.sexol.2010.09.003

[CIT0009] Bowleg, L. (2008). When black + lesbian + woman ≠ black lesbian woman: The methodological challenges of qualitative and quantitative intersectionality research. *Sex roles*, *59*, 312–325. 10.1007/s11199-008-9400-z

[CIT0010] Bowleg, L. (2013). “Once you’ve blended the cake, you can’t take the parts back to the main ingredients”: Black gay and bisexual men’s descriptions and experiences of intersectionality. *Sex roles*, *68*, 754–767. 10.1007/s11199-012-0152-4

[CIT0011] Bradford, N. J., DeWitt, J., Decker, J., Berg, D. R., Spencer, K. G., & Ross, M. W. (2019). Sex education and transgender youth: ‘Trust means material by and for queer and trans people’. *Sex Education*, *19*(1), 84–98. 10.1080/14681811.2018.1478808

[CIT0012] Bradford, N. J., Rider, G. N., & Spencer, K. G. (2021). Hair removal and psychological well-being in transfeminine adults: Associations with gender dysphoria and gender euphoria. *Journal of Dermatological Treatment*, *32*(6), 635–642. 10.1080/09546634.2019.1687823 31668100

[CIT0013] Bradford, N. J., & Spencer, K. (2020). Sexual pleasure in transgender and gender diverse individuals: An update on recent advances in the field. *Current Sexual Health Reports*, *12*(4), 314–319. 10.1007/s11930-020-00284-2

[CIT0014] Braun, V., & Clarke, V. (2006). Using thematic analysis in psychology. *Qualitative Research in Psychology*, *3*(2), 77–101. 10.1191/1478088706qp063oa

[CIT0015] Braun, V., & Clarke, V. (2019). Reflecting on reflexive thematic analysis. *Qualitative Research in Sport, Exercise and Health*, *11*(4), 589–597. 10.1080/2159676X.2019.1628806

[CIT0016] Braun, V., & Clarke, V. (2021). One size fits all? What counts as quality practice in (reflexive) thematic analysis? *Qualitative Research in Psychology*, *18*(3), 328–352. 10.1080/14780887.2020.1769238

[CIT0017] Braun, V., & Clarke, V. (2023). Toward good practice in thematic analysis: Avoiding common problems and be(com)ing a knowing researcher. *International Journal of Transgender Health*, *24*(1), 1–6. 10.1080/26895269.2022.2129597 PMC9879167 36713144

[CIT0018] Braun, V., & Clarke, V. (2024). Supporting best practice in reflexive thematic analysis reporting in palliative medicine: A review of published research and introduction to the reflexive thematic analysis reporting guidelines (rtarg). *Palliat Med*, *38*(6), 608–616. 10.1177/02692163241234800 PMC11157981 38469804

[CIT0019] Brookfield, S., Dean, J., Forrest, C., Jones, J., & Fitzgerald, L. (2020). Barriers to accessing sexual health services for transgender and male sex workers: A systematic qualitative meta-summary. *AIDS and Behavior*, *24*(3), 682–696. 10.1007/s10461-019-02453-4 30868447

[CIT0020] Byrne, D. (2022). A worked example of braun and clarke’s approach to reflexive thematic analysis. *Quality & Quantity*, *56*(3), 1391–1412. 10.1007/s11135-021-01182-y

[CIT0021] Cannoot, P. (2019). ‘#wontbeerased’: The effects of (de)pathologisation and (de)medicalisation on the legal capacity of trans* persons. *International Journal of Law and Psychiatry*, *66*, 101478. 10.1016/j.ijlp.2019.101478 31706402

[CIT0022] Cass, V. C. (1979). Homosexual identity formation: A theoretical model. *Journal of homosexuality*, *4*(3), 219–235. 10.1300/J082v04n03_01 264126

[CIT0023] Cavalcante, A. (2016). “I did it all online:” transgender identity and the management of everyday life. *Critical Studies in Media Communication*, *33*(1), 109–122. 10.1080/15295036.2015.1129065

[CIT0024] Cipolletta, S., Votadoro, R., & Faccio, E. (2017). Online support for transgender people: An analysis of forums and social networks. *Health & Social Care in the Community*, *25*(5), 1542–1551. 10.1111/hsc.12448 28329916

[CIT0026] Dhejne, C., Van Vlerken, R., Heylens, G., & Arcelus, J. (2016). Mental health and gender dysphoria: A review of the literature. *International Review of Psychiatry*, *28*(1), 44–57. 10.3109/09540261.2015.1115753 26835611

[CIT0027] Ford, J. V., Corona-Vargas, E., Cruz, M., Fortenberry, J. D., Kismodi, E., Philpott, A., Rubio-Aurioles, E., & Coleman, E. (2021). The world association for sexual health’s declaration on sexual pleasure: A technical guide. *International Journal of Sexual Health*, *33*(4), 612–642. 10.1080/19317611.2021.2023718 PMC10903694 38595778

[CIT0028] Galupo, M. P., Pulice-Farrow, L., Clements, Z. A., & Morris, E. R. (2020). 197 “i love you as both and i love you as neither”: Romantic partners’ affirmations of nonbinary trans individuals. In *Non-binary and genderqueer genders* (pp. 197–209). Routledge. 10.1080/15532739.2018.1496867 PMC6831023 32999616

[CIT0029] Gamarel, K. E., Reisner, S. L., Laurenceau, J.-P., Nemoto, T., & Operario, D. (2014). Gender minority stress, mental health, and relationship quality: A dyadic investigation of transgender women and their cisgender male partners. *Journal of Family Psychology*, *28*(4), 437. 10.1037/a0037171 PMC4122619 24932942

[CIT0030] Gamarel, K. E., Sevelius, J. M., Reisner, S. L., Coats, C. S., Nemoto, T., & Operario, D. (2018). Commitment, interpersonal stigma, and mental health in romantic relationships between transgender women and cisgender male partners. *Journal of Social and Personal Relationships*, *36*(7), 2180–2201. 10.1177/0265407518785768 PMC6510026 31086428

[CIT0031] Gillespie, I. J., Armstrong, H. L., & Ingham, R. (2022). Exploring reflections, motivations, and experiential outcomes of first same-sex/gender sexual experiences among lesbian, gay, bisexual, and other sexual minority individuals. *The Journal of Sex Research*, *59*(1), 26–38. 10.1080/00224499.2021.1960944 34406093

[CIT0032] Gowen, L. K., & Winges-Yanez, N. (2014). Lesbian, gay, bisexual, transgender, queer, and questioning youths’ perspectives of inclusive school-based sexuality education. *The Journal of Sex Research*, *51*(7), 788–800. http://www.jstor.org/stable/43701776 10.1080/00224499.2013.806648 24003908

[CIT0033] Goyal, R., Chandran, D., Garg, K., Mohankumar, P., Gupta, S., Gautam, R. K., Chopra, H., & Dhama, K. (2022). Medicalization of sexuality and sexual health: A per­spective review. *Journal of Experimental Biology and Agricultural Sciences*, *10*(6), 1241–1252. 10.18006/2022.10(6).1241.1252

[CIT0034] Griffiths, D. A., & Armstrong, H. L. (2024). “They were talking to an idea they had about me”: A qualitative analysis of transgender individuals’ experiences using dating apps. *The Journal of Sex Research*, *61*(1), 119–132. 10.1080/00224499.2023.2176422 36799719

[CIT0035] Haley, S. G., Tordoff, D. M., Kantor, A. Z., Crouch, J. M., & Ahrens, K. R. (2019). Sex education for transgender and non-binary youth: Previous experiences and recommended content. *J Sex Med*, *16*(11), 1834–1848. 10.1016/j.jsxm.2019.08.009 31585806

[CIT0036] Hall, T., Rees, C., & Newnham, E. (2024). Pathways to euphoria: Deconstructing gender-expansive adults conceptualizations of wellbeing. *International Journal of Applied Positive Psychology*, 9(2), 719-745. 10.1007/s41042-024-00149-2

[CIT0037] Hendricks, M. L., & Testa, R. J. (2012). A conceptual framework for clinical work with transgender and gender nonconforming clients: An adaptation of the minority stress model. *Professional Psychology: Research and Practice*, *43*, 460–467. 10.1037/a0029597

[CIT0038] Hobaica, S., Schofield, K., & Kwon, P. (2019). “Here’s your anatomy…good luck”: Transgender individuals in cisnormative sex education. *American Journal of Sexuality Education*, *14*(3), 358–387. 10.1080/15546128.2019.1585308

[CIT0039] Holmberg, M., Arver, S., & Dhejne, C. (2019). Supporting sexuality and improving sexual function in transgender persons. *Nature Reviews Urology*, *16*(2), 121–139. 10.1038/s41585-018-0108-8 30375495

[CIT0040] Iantaffi, A., & Bockting, W. O. (2011). Views from both sides of the bridge? Gender, sexual legitimacy and transgender people’s experiences of relationships. *Culture, Health & Sexuality*, *13*(3), 355–370. 10.1080/13691058.2010.537770 PMC3076785 21229422

[CIT0041] Jacobsen, K., & Devor, A. (2022). Moving from gender dysphoria to gender euphoria: Trans experiences of positive gender-related emotions. *Bulletin of Applied Transgender Studies*, *1*(1-2), 119–143. 10.57814/ggfg-4j14

[CIT0042] Jessani, A., Berry-Moreau, T., Parmar, R., Athanasakos, A., Prodger, J. L., & Mujugira, A. (2024). Healthcare access and barriers to utilization among transgender and gender diverse people in africa: A systematic review. *BMC Global and Public Health*, *2*(1), 44. 10.1186/s44263-024-00073-2 PMC11208260 38948028

[CIT0043] Johnson, A. H. (2019). Rejecting, reframing, and reintroducing: Trans people’s strategic engagement with the medicalisation of gender dysphoria. *Sociology of Health & Illness*, *41*(3), 517–532. 10.1111/1467-9566.12829 30484870

[CIT0044] Jun, H. (2018). Cissexism (genderism or binarism). In H. Jun (Ed.), *Social justice, multicultural counseling, and practice: Beyond a conventional approach* (pp. 161–185). Springer International Publishing. 10.1007/978-3-319-72514-7_6

[CIT0045] Kılıç Onar, D., Armstrong, H., & Graham, C. A. (2020). What does research tell us about women’s experiences, motives and perceptions of masturbation within a relationship context?: A systematic review of qualitative studies. *Journal of Sex & Marital Therapy*, *46*(7), 683–716. 10.1080/0092623X.2020.1781722 32628093

[CIT0046] Laan, E. T. M., Verena, K., A., W. M., W., v. L. R. H., & and Janssen, E. (2021). In pursuit of pleasure: A biopsychosocial perspective on sexual pleasure and gender. *International Journal of Sexual Health*, *33*(4), 516–536. 10.1080/19317611.2021.1965689 PMC10903695 38595780

[CIT0047] Lev, A. I. (2004). *Transgender emergence: Understanding diverse gender identities and expressions*. Haworth Clinical Practice Press.

[CIT0048] Lorimer, K., DeAmicis, L., Dalrymple, J., Frankis, J., Jackson, L., Lorgelly, P., McMillan, L., & Ross, J. (2019). A rapid review of sexual wellbeing definitions and measures: Should we now include sexual wellbeing freedom? *The Journal of Sex Research*, *56*(7), 843–853. 10.1080/00224499.2019.1635565 31335208

[CIT0049] Marshall, E., Glazebrook, C., Robbins-Cherry, S., Nicholson, S., Thorne, N., & Arcelus, J. (2020). The quality and satisfaction of romantic relationships in transgender people: A systematic review of the literature. *International Journal of Transgender Health*, *21*(4), 373–390. 10.1080/26895269.2020.1765446 PMC8726697 34993516

[CIT0050] Martin, T. K., & Coolhart, D. (2022). “Because your dysphoria gets in the way of you…it affects everything.” the mental, physical, and relational aspects of navigating body dysphoria and sex for trans masculine people. *Sexual and Relationship Therapy*, *37*(1), 82–99. 10.1080/14681994.2019.1696459

[CIT0051] McGeorge, C. R., Coburn, K. O., & Walsdorf, A. A. (2021). Deconstructing cissexism: The journey of becoming an affirmative family therapist for transgender and nonbinary clients. *Journal of Marital and Family Therapy*, *47*(3), 785–802. 10.1111/jmft.12481 33493357

[CIT0052] Miller, L. R., & Grollman, E. A. (2015). The social costs of gender nonconformity for transgender adults: Implications for discrimination and health. *Sociological Forum*, *30*(3), 809–831. 10.1111/socf.12193 PMC5044929 27708501

[CIT0053] Nash, C. J., & Browne, K. (2019). Resisting the mainstreaming of LGBT equalities in Canadian and British schools: Sex education and trans school friends. *Environment and Planning C: Politics and Space*, *39*(1), 74–93. 10.1177/2399654419887970

[CIT0054] Nikkelen, S. W. C., & Kreukels, B. P. C. (2018). Sexual experiences in transgender people: The role of desire for gender-confirming interventions, psychological well-being, and body satisfaction. *Journal of Sex & Marital Therapy*, *44*(4), 370–381. 10.1080/0092623X.2017.1405303 29144853

[CIT0055] Office for National Statistics. (2022). *Ethnic group, England and wales: Census 2021*. https://www.ons.gov.uk/peoplepopulationandcommunity/culturalidentity/ethnicity/bulletins/ethnicgroupenglandandwales/census2021#cite-this-statistical-bulletin

[CIT0056] Office for National Statistics. (2023). *Gender identity, England and wales: Census 2021*. https://www.ons.gov.uk/peoplepopulationandcommunity/culturalidentity/genderidentity/bulletins/genderidentityenglandandwales/census2021

[CIT0057] Özer, M., Poor Toulabi, S., Gijs, L., Kreukels, B. P. C., & Mullender, M. G. (2022). The assessment of sexual wellbeing in treatment-seeking transgender individuals: A systematic review of the medical literature. *International Journal of Impotence Research*, 32(2), 121-131. 10.1038/s41443-022-00529-9 35115682

[CIT0058] Pandya, A. k., & Redcay, A. (2021). Access to health services: Barriers faced by the transgender population in India. *Journal of Gay & Lesbian Mental Health*, *25*(2), 132–154. 10.1080/19359705.2020.1850592

[CIT0059] Platt, L. F. (2020). An exploratory study of predictors of relationship commitment for cisgender female partners of transgender individuals. *Family Process*, *59*(1), 173–190. 10.1111/famp.12400 30311216

[CIT0060] Puckett, J. A., Aboussouan, A. B., Ralston, A. L., Mustanski, B., & Newcomb, M. E. (2023). Systems of cissexism and the daily production of stress for transgender and gender diverse people. *International Journal of Transgender Health*, *24*(1), 113–126. 10.1080/26895269.2021.1937437 PMC9879165 36713141

[CIT0061] Pulice-Farrow, L., Bravo, A., & Galupo, M. P. (2019). “Your gender is valid”: Microaffirmations in the romantic relationships of transgender individuals. *Journal of LGBT Issues in Counseling*, *13*(1), 45–66. 10.1080/15538605.2019.1565799

[CIT0062] QSR International Pty Ltd. (2020). Nvivo. https://www.qsrinternational.com/nvivo-qualitative-data-analysis-software/home

[CIT0064] Ross, K. A., & Scholl, J. C. (2016). Socially supported transition: How transgender individuals use the internet to navigate medical transition. *Iowa Journal of Communication*, *48*(1-2), 35–38.

[CIT0065] Rubin, J. D., Conley, T. D., Klein, V., Liu, J., Lehane, C. M., & Dammeyer, J. (2019). A cross-national examination of sexual desire: The roles of ‘gendered cultural scripts’ and ‘sexual pleasure’ in predicting heterosexual women’s desire for sex. *Personality and Individual Differences*, *151*, 109502. 10.1016/j.paid.2019.07.012

[CIT0066] Smith, K. P., Madison, C. M., & Milne, N. M. (2014). Gonadal suppressive and cross-sex hormone therapy for gender dysphoria in adolescents and adults. *Pharmacotherapy: The Journal of Human Pharmacology and Drug Therapy*, *34*(12), 1282–1297. 10.1002/phar.1487 25220381

[CIT0067] Stephenson, R., Riley, E., Rogers, E., Suarez, N., Metheny, N., Senda, J., Saylor, K. M., & Bauermeister, J. A. (2017). The sexual health of transgender men: A scoping review. *The Journal of Sex Research*, *54*(4-5), 424–445. 10.1080/00224499.2016.1271863 28140660

[CIT0068] Tan, K. K. H., Yee, A., & Veale, J. F. (2021). “Being trans intersects with my cultural identity”: Social determinants of mental health among asian transgender people. *Transgender Health*, *7*(4), 329–339. 10.1089/trgh.2021.0007 PMC9398473 36033212

[CIT0070] Waldura, J. F., Arora, I., Randall, A. M., Farala, J. P., & Sprott, R. A. (2016). Fifty shades of stigma: Exploring the health care experiences of kink-oriented patients. *The Journal of Sexual Medicine*, *13*(12), 1918–1929. 10.1016/j.jsxm.2016.09.019 28340946

[CIT0071] Westbrook, L. (2021). *Unlivable lives: Violence and identity in transgender activism/laurel Westbrook*. University of California Press.

[CIT0072] White, C. R., & Jenkins, D. D. (2017). College students’ acceptance of trans women and trans men in gendered spaces: The role of physical appearance. *Journal of Gay & Lesbian Social Services*, *29*(1), 41–67. 10.1080/10538720.2016.1261749

[CIT0073] Whitney, N., Samuel, A., Douglass, L., Strand, N. K., & Hamidian Jahromi, A. (2022). Avoiding assumptions: Sexual function in transgender and non-binary individuals. *The Journal of Sexual Medicine*, *19*(6), 1032–1034. 10.1016/j.jsxm.2022.03.214 35466068

[CIT0074] World Health Organization. (2022). *International statistical classification of diseases and related health problems* (11 ed.). World Health Organization. https://icd.who.int/10.2471/BLT.25.294650 PMC13531092 42683092

[CIT0075] Wurm, M., T., L., Mejias, N. T., & and Malmquist, A. (2024). Minority joy, minority growth, and minority peace: Transgender and gender diverse people’s varied positive lived experiences. *International Journal of Transgender Health*, 1–16. 10.1080/26895269.2024.2445094 PMC12857694 41625004

